# Effect of postpartum pessary use on pelvic floor function: a prospective multicenter study

**DOI:** 10.1007/s00404-024-07758-x

**Published:** 2024-10-10

**Authors:** Franziska Beer, Madeleine Kuppinger, Frank Schwab, Markus Hübner, Brenda Kiefner, Anna Nacke, Ute Kelkenberg, Sabine Schütze, Anna Lindner, Lars Hellmeyer, Wolfgang Janni, Melanie Metz, Miriam Deniz

**Affiliations:** 1 Department of Obstetrics and Gynecology, University Hospital of Ulm, Ulm, Germany; 2 Institute of Hygiene and Environmental Medicine, Charité Universitätsmedizin Berlin, Berlin, Germany; 3 Department of Obstetrics and Gynecology, University Hospital of Freiburg, Freiburg, Germany; 4 Department of Obstetrics, Vivantes Klinikum Friedrichshain, Berlin, Germany; 5 Departement of Obstetrics and Gynecology, Klinikum Bielefeld, Bielefeld, Germany; 6 Department of Sexual Health and Family Planning, Berlin, Germany

**Keywords:** Restifem® pessary, Pelvic floor dysfunction, Postpartum pelvic floor therapy, German pelvic floor questionnaire for pregnant and postpartum women

## Abstract

**Purpose:**

This study evaluates the restitution of pelvic floor function in postpartum women using the Restifem® pessary in a preventive and therapeutic approach.

**Methods:**

In this multicentre study all postpartum women independently of their parity, mode of delivery and existing pelvic floor symptoms were offered to use the Restifem® pessary from 6 weeks postpartum for 3 to 6 months. They completed the validated German pelvic floor questionnaire (GPFQpp) via online survey at 6 weeks, 6 months and 12 months postpartum and were divided, by their own choice, into users and non-users of the pessary.

**Results:**

Initially 857 women were enrolled. After 6 weeks 137 pessary users and 133 non-users, after 12 months 53 pessary users and 45 non-users submitted a completed questionnaire. Pessary users had significantly higher (worse) scores in all domains of the GPFQpp at 6 weeks postpartum. At 12 months postpartum pessary users still had a significantly higher bladder score, compared to non-users. There was a greater improvement in the bladder score (p = 0.005) and the pelvic organ prolapse score (p < 0.001) from 6 weeks to 12 months postpartum, among pessary users compared to non-users.

**Conclusion:**

Pessary users had a significantly greater improvement in pelvic floor function from 6 weeks to 12 months postpartum, compared to non-users. This effect might be in part due to wearing the pessary but also due to greater scope for recovery, given the higher level of pelvic floor dysfunction in the pessary user group.

**Trial registration.:**

The trial was registered in the German Clinical Trials Register (DRKS00024733) on 19 of April 2021.

## What does this study add to clinical work

**Table:** 

This prospective study evaluates the effect of the Restifem® pessary, developed for pelvic floor restitution in postpartum women by using the validated German pelvic floor questionnaire for pregnant and postpartum women (GPFQpp). Pessary users had higher (worse) scores in pelvic floor function and over time an increased improvement in pelvic floor symptoms compared to women not using the pessary after 12 months postpartum.	

## Introduction

Pelvic floor dysfunction, such as urinary incontinence, faecal incontinence and pelvic organ prolapse (POP), affects approximately 30% of all women [1, 2]. Pregnancy and childbirth are considered primary risk factors for these conditions. The literature reports a prevalence of urinary incontinence of 7–36% at 6–13 weeks postpartum, and 11–51% at 12 months postpartum [3]. Pelvic floor muscle training (PFMT) reduces the occurrence of urinary incontinence, during pregnancy and postpartum. However, the effect of postpartum PFMT for preventing and treating urinary incontinence or prolapse is limited [4–6].

The cause of postpartum pelvic floor dysfunction is the trauma to muscle, nerve, and connective tissue [7–9]. To facilitate the healing of muscle and connective tissue after injury, early mobilization and stabilization with medical devices is a common therapeutic approach in orthopaedics [10]. There are two small, prospective studies evaluating the effect of postpartum pessary use on incontinence and POP showing a reduction in incontinence—and POP symptoms [11, 12].

The Restifem® (Restitutio feminina) pessary was developed specifically for postpartum women and is approved for the treatment of postpartum POP and stress urinary incontinence (SUI). Its shape is precisely adapted to the female pelvic floor, therefore it strengthens the levels of support along the lateral vaginal wall (Level II), the uterus (Level I) and, suburethral, the anterior vaginal wall (Level III) [13, 14]. It is thereby relieving pressure on the muscular and connective tissue structures of the pelvic floor.

We confirmed the applicability of Restifem® pessaries, by showing that the use of the pessary could reduce symptoms of prolapse, urinary incontinence and overactive bladder (OAB) 6 months postpartum. The primary outcome of this published study was applicability and compliance and so the questionnaire was not validated for assessment of pelvic floor disorders [15].

Therefore, the objective of the present analysis was to compare the restitution of pelvic floor function in women using the Restifem® pessary in comparison to women not using the pessary. Women received the pessary independently of pelvic floor symptoms, thus enabling us to investigate its therapeutic and preventive effect. It was the hypothesis of this trial, that symptomatic women were more likely to use the pessary and that their improvement of symptoms would be greater that non-users.

## Methods

The study was approved by local ethics committees and registered at the DRKS (DRKS00024733).

### Study population

The study was conducted in three tertiary care hospitals in Germany in 2021. Inclusion criteria were age over 18 years, to command German language and given informed consent. Exclusion criteria were inflammation of the vagina, allergy to silicone or not being able to handle the pessary. Women were included on the 1st/2nd day postpartum and pessary was handed out (after caesarean section “small” and after vaginal “medium” Restifem® pessary) [15]. After 6 weeks postpartum participants received an email, reminding them to start wearing the pessary, along with a link to an instructional video.

### Restifem®

The Restifem® pessary is made of a tissue-compatible silicone, and its shape is adapted to the anatomy of the vagina. It was developed specifically for women postpartum, the manufacturer´s recommendation being to start using it at 6 weeks postpartum and to continue use for 3 to 6 months following the birth.

### Online survey

LimeSurvey® Software versions 3.27.4 was used at intervals of 6 weeks, 6 months and 12 months, during which time postpartum participants received the GPFQpp online. Additionally, women answered the question, whether they still use the pessary [15].

### Primary and secondary endpoints

The primary endpoint of the study was the change in the four scores of the validated GPFQpp from 6 weeks postpartum to 12 months postpartum in pessary users compared to non-users. Secondary objectives were the impact of the pessary on symptoms and distress of pelvic floor dysfunction.

### Questionnaire

The validated German Pelvic Floor Questionnaire for pregnant and postpartum women (GPFQ p) comprises the domains of bladder-, bowel-, prolapse- and sexual function (domain score 0–10; total score 0–40) and evaluates the impact on quality of life during pregnancy and postpartum (QoL domain score 0–10) [16].

### Statistical analysis

To answer the primary question, we compared the individual scores of the 4 domains of the GPFQpp 6 weeks postpartum and 12 months postpartum between pessary users and non-users. Twelve women reported still wearing the pessary after 12 months postpartum; these were not included in the analysis to avoid potential influence of the pessary usage on the results. To calculate the improvement of scores over time, from 6 weeks postpartum to 12 months postpartum, the difference between the score at 12 months postpartum and 6 weeks postpartum was computed for each group. To address the question of which factors influence pessary usage, we analysed the data 6 weeks postpartum, from women who still completed the questionnaire 6 months postpartum.

In the descriptive analysis, number and percent’s, mean with standard deviation (SD) and median and interquartile range (IQR) were calculated depending of distribution of parameters. Differences between groups were tested using Chi-square test or Mann–Whitney U-test. Differences within individuals were tested using Wilcoxon rank sum test for paired sample.

All analyses were exploratory in nature and performed with IBM SPSS statistics version 29.0 (Armonk, New York, United States).

## Results

### Differences in pelvic floor function 6 weeks postpartum between pessary users and non- users

Pessary users were those who reported wearing the pessary at least at one of the four time points 8 weeks, 3 months, and/or 6 months postpartum. Non-users consisted of those who initially opted against pessary use and those who initially chose pessary use, but reported at 8 weeks postpartum not wearing it [15].

**Fig. 1:**
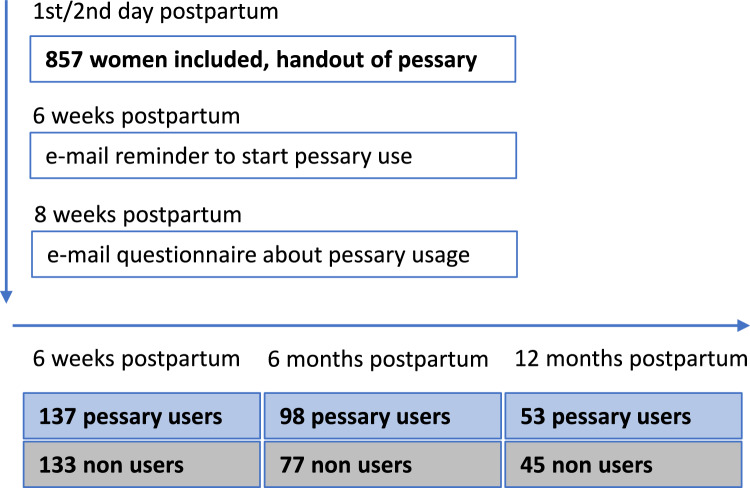
Time table and return rate of evaluable questionnaires

Initially 857 women were enrolled on the 1st/2nd day postpartum with a high drop-out rate at 6 weeks, 6 months and 12 months postpartum (Fig. 1).

In the 6 months postpartum observed group among the pessary users significantly more women reported having a family history of pelvic floor dysfunction, compared to non-users (31.6% versus 11.7%; p = 0.007). There was no significant difference between the groups in the areas of weight, age, number of births, birth weight of the heaviest child and higher-grade perineal tears.

#### Scores

Women who used the pessary had significantly higher (worse) scores in the bladder, bowel, POP and sexual domain of the GPFQpp, 6 weeks postpartum, compared to the non-users (Table 1).

**Table 1:** Scores of the German Pelvic Floor Questionnaire for pregnant and postpartum women after 6 weeks postpartum of pessary users and non-users in n = 175 (98 pessary users and 77 non-users) women who still completed the questionnaire 6 months postpartum

Score of the pelvic floor questionnaire	Pessary users Median (IQR)	Non users Median (IQR)	p value
Bladder Score	1.25 (0.83–2.29)	0.89 (0.63–1.46)	**0.005**
Bowel Score	1.94 (0.97–2.58)	1.29 (0.97–1.94)	**0.001**
Pelvic organ prolapse Score	0 (0–2)	0 (0–0.67)	**0.038**
Sex Score	1.67 (0.74–2.96)	1.11 (0.37–2.22)	**0.044**

Bold value indicates p < 0.05

p-value, Wilcoxon rank-sum test, *SD* Standard deviation, *Iqr* Interquartile range, *N* Number, min, Minimum, max, Maximum

#### Symptoms

Symptomatic women were defined as those who reported in the GPFQpp symptoms of SUI, OAB, or POP. Women with SUI were defined as those who answered the question “Do you leak with coughing, sneezing, laughing or exercising?” with: “occasionally (2)”, “frequently (3) or ”daily “(4). Women OAB were defined as those who answered the question S4 “Do you need to rush/hurry to pass urine when you get the urge? ” and/or S5 “Does urine leak when you rush or hurry to the toilet or can’t you make it in time?” with 2,3, or 4. Women with symptoms of POP were defined as those who answered one of the following questions with 2,3, or 4: “U1 Do you have a sensation of tissue protrusion/lump/bulging in your vagina?”, U2 “Do you experience vaginal pressure or heaviness or a dragging sensation?” or “U3 Do you have a feeling of vaginal or uterus prolapse when lifting, walking, or running?”.

Pessary users reported significantly higher rates of SUI at 6 weeks postpartum. There were no significant differences in symptoms of an OAB or POP (Table 2).

**Table 2:** Symptoms and distress reported in the German Pelvic Floor Questionnaire for pregnant and postpartum women after 6 weeks postpartum of pessary users and non-users in N = 175 women who still completed the questionnaire 6 months postpartum

Symptoms reported in the pelvic floor questionnaire	Pessary users (N = 98)	Non-users (N = 77)	P value
Stress urinary incontinence (n (%))	54 (55.1%)	30 (39.5%)	**0.041**
Overactive bladder (n (%))	63 (64.3%)	39 (50.6%)	0.069
Pelvic organ prolapse (n (%))	43 (45.3%)	24 (31.2%)	0.059
Distress reprtoed in the pelvic floor questionnaire due to incontinence and/or pelvic organ prolapse (n (%))	57 (58.2%)	31 (40.3%)	**0.019**

Bold values indicate p < 0.05

#### Distress

Women with distress were those who indicated in the GPFQpp that urinary loss and/or symptoms of prolapse affected their daily life and/or reported finding the symptoms of prolapse bothersome. We defined distress due to SUI when women answered the question “Does urine loss affect your daily life? (e.g., sports, work, shopping, socializing) “(S15) with ”a little “ (3), ”quite a bit “ (4), ”very much “ (5). We defined distress due to POP when women answered one of the following questions with 3,4, or 5: ”Does prolapse affect your daily life? “ (U4) and or ”How much does your prolapse bother you? “ (U5).”

In the group of pessary users, there was a significantly higher level of distress due to incontinence and/or prolapse at 6 weeks postpartum, compared to non-users (Table 2).

### Changes in pelvic floor function 12 months postpartum in pessary users vs non-users

#### Scores

After 12 months, N = 98 women completed the questionnaire. The pessary users still had a significantly worse bladder score compared to non-users. However, there was no significant difference anymore in the bowel-, sex- or POP score between the two groups (Table 3).

**Table 3:** Scores of the German Pelvic Floor Questionnaire for pregnant and postpartum women after 12 months postpartum of pessary users and non-users in N = 98 women who still completed the questionnaire 12 months postpartum

Score of the pelvic floor questionnaire	Pessary users (N)	Non users	P value
	Median (IQR)	Median (IQR)	
Bladder score	0.95 (0.44–2)	0.83 (0.42–1.11)	**0.048**
Bowel score	0 (0–0.67)	1.29 (0.65–1.7)	0.254
Pelvic organ prolapse score	0 (0–0.67)	0 (0–0.67)	0.307
Sex score	0.89 (0.37–1.85)	0.37 (0–1.48)	0.051

Bold value indicates p < 0.05

**Fig. 2:**
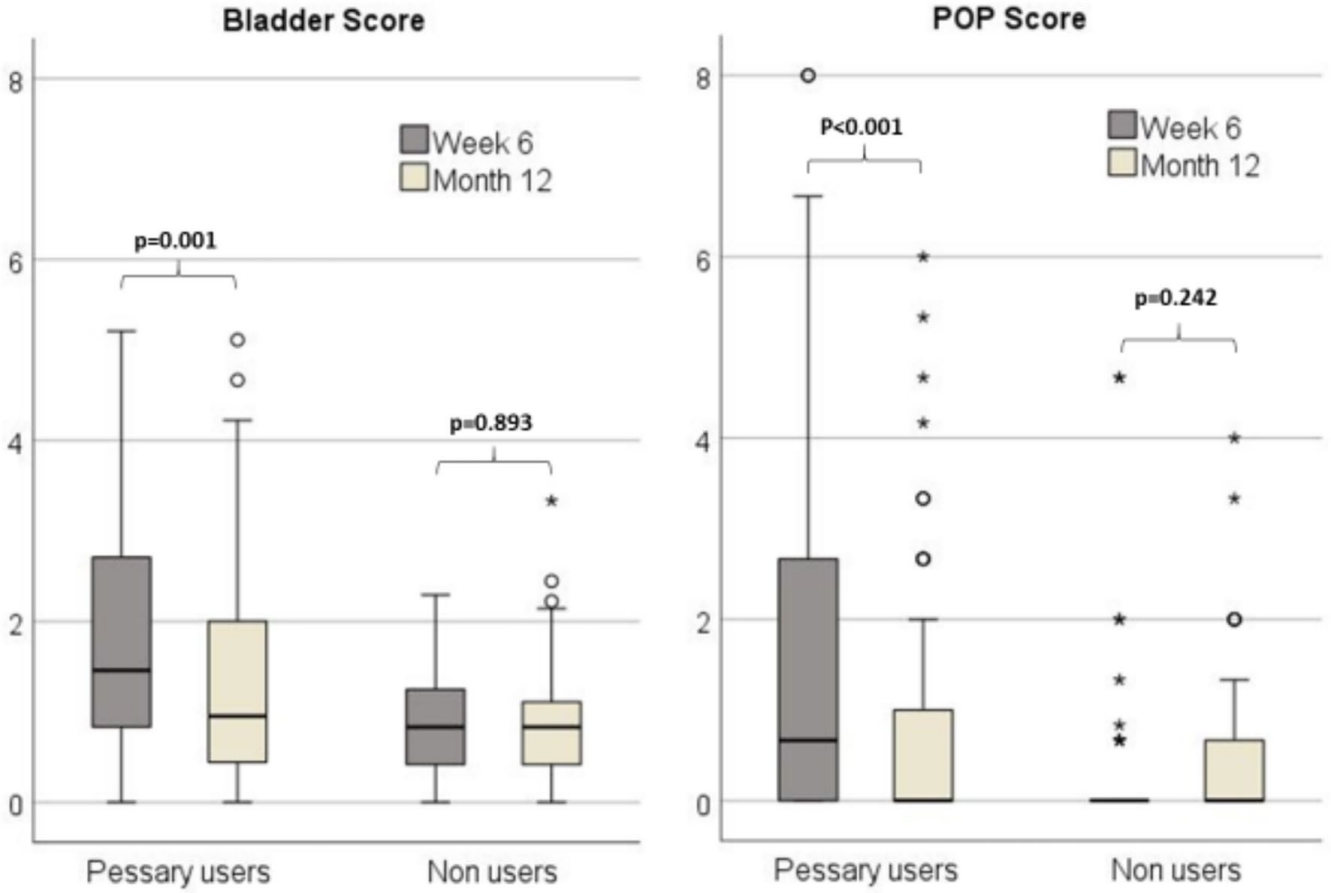
Score value of the bladder score and pelvic organ prolapse score of the German Pelvic Floor Questionnaire for pregnant and postpartum women 6 months and 12 months postpartum of pessary users and non-users in N = 98 women who still completed the questionnaire 12 months postpartum Significance was tested with Wilcoxon ranksum test for paired sample

The higher the score, the more symptoms exist. Therefore, the improvement is greater the smaller the value of the difference. A negative value implies an improvement and a positive value an increase in symptoms. There was a greater improvement in the bladder score and the POP score among the pessary users compared to the non-users (Table 4 and Fig. 2). Regarding the improvement over time in one group, pessary users had significantly lower bladder and POP scores at 12 months postpartum compared to 6 months postpartum, whereas the improvement among non-users was not significant (Fig. 2).

**Table 4:** Difference 6 weeks to 12 months postpartum of score and symptoms in the German Pelvic Floor Questionnaire for pregnant and postpartum women of pessary users and non-users

	Difference 6 weeks to 12 months postpartum Pessary users	Difference 6 weeks to 12 months postpartum Non users	P value
	Median (IQR)	Median (IQR)	
Score of the pelvic floor questionnaire			
Bladder score	−0.36 (−0.69–0)	0 (−0.42–0.4)	**0.005**
Bowel score	−0.65 (−1.29–0)	−0.07 (−0.65–0.32)	0.060
Pelvic organ prolapse score	0 (−2–0)	0 (0–0.67)	**0.001**
Sex score	−0.37 (−2.22–0.24)	−0.58 (−1.3–0.07)	0.694
Symptoms reported in the pelvic floor questionnaire			
Stress urinary incontinence	0 (0–0)	0 (0–0)	0.154
Overactive bladder (S4)	0 (−1–0)	0 (0–0)	0.451
Overactive bladder (S5)	0 (0–0)	0 (0–0)	0.237
Pelvic organ prolapse (U1)	0 (0–0)	0 (0–0)	**0.04**
Pelvic organ prolapse (U2)	0 (−1–0)	0 (0–0)	**0.039**
Pelvic organ prolapse (U3)	0 (−1–0)	0 (0–0)	**0.005**

Bold values indicate p < 0.05

#### Symptoms

Pessary users had significantly higher rates of SUI and OAB after 12 months postpartum compared to non-users. There was no significant difference in terms of symptoms of POP (Table 5). When calculating the improvement there was no significant difference in improvement concerning symptoms due to SUI and OAB between the two groups. However, there was a greater improvement over time of symptoms of POP regarding the questions U1, U2 and U3 of the GPFQpp among pessary users compared to non-users (Table 4).

**Table 5:** Symptoms and distress reported in the German Pelvic Floor Questionnaire for pregnant and postpartum women after 12 months postpartum of pessary users and non-users

	Pessary users (N = 53)	Non-users (N = 45)	P Vlaue
*Symptoms reported in the pelvic floor questionnaire*			
Stress urinary incontinence (n (%))	33 (66.3%)	17 (39.5%)	**0.027**
Overactive bladder (n (%))	29 (54.7%)	15 (34.1%)	**0.042**
Pelvic organ prolapse (n (%))	24 (45.3%)	15 (35.7%)	0.346
Distress in the pelvic floor questionnaire due to incontinence and/or pelvic organ prolapse (n (%))	30 (56.6%)	17 (38.6%)	0.078

Bold values indicate p < 0.05

#### Distress

There was no significant difference in terms of level of distress due to incontinence and/or prolapse 12 months postpartum among pessary users compared to non-users (Table 5).

## Discussion

The aim of our study was to evaluate the effect of the Restifem® pessary in reconstitution of pelvic floor function postpartum in a preventive and therapeutic approach.

### Therapy of pelvic floor dysfunction postpartum

Pelvic floor disorders postpartum are common and risk factors such as age at first delivery and obesity are increasing [3]. During labour trauma to muscle, nerve, and connective tissue can lead to pelvic floor disorders [7–9] Although these symptoms are common, not all women find them bothersome. Therefore, it is important to identify those who suffer and implement an appropriate therapy for those women [17]. Treating and preventing urinary incontinence or POP with PFMT is limited [4–6, 18]. In contrast to PFMT, the therapeutic approach of the Restifem® pessary focuses on stability which is a common therapy in orthopaedic disease [10]. For this reason, the Restifem® pessary provides us with an exciting opportunity for a new approach in the therapy and prevention of postpartum pelvic floor disorder. So far there are two small, prospective studies evaluating the effect of postpartum pessary use on incontinence and POP showing a reduction in incontinence and POP symptoms [11, 12].

### Motivational factors

Alewijnse et al. describe determinants of adherence to pelvic floor muscle exercise for women with urine incontinence. Beliefs and illness as well as outcome expectations and perceived severity of symptoms were determinants for adherence [20]. In our study, 6 weeks postpartum, the group of pessary users showed significantly higher (worse) scores in all 4 domains of the GPFQpp, more frequent symptoms of SUI and a higher level of distress regarding incontinence and prolapse. These parameters could be interpreted as illness representation and perceived severity of symptoms and thus determinants for using the pessary.

According to the Attitude Social Influence Self-Efficacy (ASE) model variables such as sociodemographic and psychosocial variables are expected to influence behavioural intention [21, 22]. In the group of pessary users, 6 weeks postpartum, a significantly higher frequency of family history for pelvic floor dysfunction was observed, underlining the sociodemographic and psychosocial variables described in the ASE, that may have led to use the pessary.

### Prevalence and progression over time of symptoms of urinary incontinence and POP postpartum

The prevalence of urinary incontinence is relatively heterogeneous. In the literature a prevalence of 7–36% at 6–13 weeks postpartum and 11–51% at 12 months postpartum is reported [3]. Our data in comparison show a higher prevalence of SUI at 6 weeks postpartum for pessary users (55.1%) and non-users (39.5%). At 12 months postpartum pessary users still had a higher prevalence (66.3%) whereas non-users were in line with the above mentioned data (39.5%). This might be due to the fact that our study design motivated especially symptomatic women to take part. Regarding the improvement of symptoms of urinary incontinence 6 weeks to 12 months postpartum, there is no significant difference between both groups of our study population. But at 12 months postpartum pessary users still had a significantly higher frequency of symptoms of SUI (62.3% versus 39.5%) and OAB (54.7% versus 34.1%) compared to non-users.

Reimers et al. investigated the prevalence of POP in a cohort of primiparous women up to one year postpartum. They found a 9% prevalence of stage II POP at 6 weeks postpartum and 2% at 12 months postpartum [23]. Another cohort study of primiparous women also showed a decrease in the occurrence of stage II POP in the first year postpartum [24]. In our study, we observed a higher prevalence of POP at 6 weeks and 12 months postpartum. There was no objective examination as in the aforementioned studies and about half of our cohort were multiparous. Our data demonstrate a significant improvement in symptoms of POP from 6 weeks to 12 months postpartum in pessary users compared to non-users.

### German pelvic floor questionnaire for pregnant and postpartum women

Metz et al. developed and validated a questionnaire for the assessment of pelvic floor disorders postpartum. They included nulliparous women who completed the questionnaire in the 3rd trimester, at 6 weeks, and 12 months postpartum. In this cohort at 12 months postpartum the bladder (1.07) and the POP score (0.86) was higher (worse) compared to 6 weeks postpartum (0.93, 0.76) but not significantly [16]. In our study at 6 weeks non-users had similar mean scores in the bladder (1.1) and POP (0.7) domain, as well as no significant improvement over time, whereas pessary users had higher scores and a significant improvement over the aforementioned time period in the bladder and POP domain. In group comparison pessary users had a significantly greater improvement from 6 weeks to 12 months postpartum, compared to non-users in the bladder and POP score, most likely to a higher level of symptoms in the beginning. But still 12 months postpartum pessary users had significantly higher scores in the bladder domain compared to non-users. Baessler et al. evaluated the minimal important difference (MID) which indicates the change in scores that is meaningful to patients [25]. A change of approximately 1 in the scores of the individual domains represents an important difference. In our cohort, in none of the four domains a change in scores of ≥ 1 is reached from 6 weeks to 12 months postpartum among pessary users or non-users. The authors state, that their findings might be specific to the cohort, undergoing an operative therapy, and smaller changes in scores, especially after conservative therapy, could also be significant [25]. Probably the most important factor in describing the impact of pelvic floor disorders on women’s quality of life is distress due to pelvic organ symptoms. Metz et al. found that for women with distress postpartum the score of the bladder and POP domain was significantly higher than for those without distress, with a difference in the bladder score of 1.1 and POP score of 3.3 [16]. These differences correspond to the MID. In our study pessary users still had a higher, but not anymore significantly relevant level of distress due to incontinence and/or prolapse 12 months postpartum compared to non-users.

### Conservative pelvic floor therapy postpartum and normal reparative process over time

Two randomised prospective trails investigating PFMT in postpartum women found an improvement of pelvic floor function postpartum in the intervention as well as in the control group with no significant difference between the groups [5, 6]. Both studies highlight the importance time in pelvic floor recovery. The pelvic floor seems weaken temporarily after childbirth, but contractility appears to recover after 1 year, irrespective of the delivery mode [26]. As our study was not primarily designed to investigate the effect of the pessary, it cannot finally be clarified whether the significantly greater improvement in the bladder and POP score, as well as in symptoms due to POP, among pessary users, is caused by wearing the pessary. Another reasonable explanation might be the greater progression over time, due to normal reparative tissue process, as in the aforementioned studies. Higher scores in the GPFGpp and a higher level of symptoms, at 6 weeks postpartum are most likely to be due to tissue trauma during labour explaining perhaps the greater improvement over time.

### Strength and limitations

A strength of our study was the prospective and multicentre design. So far this is the first prospective study using a validated questionnaire to investigating the effect of the Restifem® pessary on pelvic floor restitution postpartum, preventively and therapeutically.

As our study was not primarly designed to assess the efficacy of the pessary, we did not have randomised controlled design. Women decided themselves whether to wear the pessary, which meant no intervention and control group but users and non-users by choice. We did not exclude further confounders like PFMT. Additionally, there was a high drop-out rate.

Taken together, especially women with symptoms of pelvic floor disorder were willing to use the pessary and seem to have a benefit. Further, randomised controlled trials are needed to investigate the effect of the pessary for symptomatic women therapeutically and for asymptomatic women in a preventive intention. Additionally, assessment of the stabilising effect on the pelvic floor structures by ultrasound or MRI studies could help to better understand the recovery of pelvic floor structures.

## Conclusion

Pessary users had a significantly greater improvement in the bladder and the POP score from 6 weeks to 12 months postpartum, compared to non-users. Whether these effects are due to wearing the pessary or to greater scope for recovery, given the higher level of pelvic floor dysfunction postpartum cannot be clarified in this study. Further studies are needed to investigate the effect of pessary use postpartum.

Furthermore, symptoms and distress due to pelvic floor dysfunction alongside a family history, can be seen as motivational factors in willingness to use the pessary.
